# The Neonicotinoid Insecticide Imidacloprid Repels Pollinating Flies and Beetles at Field-Realistic Concentrations

**DOI:** 10.1371/journal.pone.0054819

**Published:** 2013-01-28

**Authors:** Amy H. Easton, Dave Goulson

**Affiliations:** Biological and Environmental Sciences, School of Natural Sciences, University of Stirling, Stirling, Scotland; Ghent University, Belgium

## Abstract

Neonicotinoids are widely used systemic insecticides which, when applied to flowering crops, are translocated to the nectar and pollen where they may impact upon pollinators. Given global concerns over pollinator declines, this potential impact has recently received much attention. Field exposure of pollinators to neonicotinoids depends on the concentrations present in flowering crops and the degree to which pollinators choose to feed upon them. Here we describe a simple experiment using paired yellow pan traps with or without insecticide to assess whether the commonly used neonicotinoid imidacloprid repels or attracts flying insects. Both Diptera and Coleoptera exhibited marked avoidance of traps containing imidacloprid at a field-realistic dose of 1 µg L^−1^, with Diptera avoiding concentrations as low as 0.01 µg L^−1^. This is to our knowledge the first evidence for any biological activity at such low concentrations, which are below the limits of laboratory detection using most commonly available techniques. Catch of spiders in pan traps was also slightly reduced by the highest concentrations of imidacloprid used (1 µg L^−1^), but catch was increased by lower concentrations. It remains to be seen if the repellent effect on insects occurs when neonicotinoids are present in real flowers, but if so then this could have implications for exposure of pollinators to neonicotinoids and for crop pollination.

## Introduction

Declines in pollinator abundance have led to fears of a ‘pollination crisis’ which threatens both agricultural productivity and biodiversity [1], [2]. Worldwide, ∼1,500 crops require insect pollination, and ∼35% of human food depends directly or indirectly on pollinators, primarily insects, with the pollination services they provide contributing an estimated $14.6 billion to the economy of the USA and £440 million/yr to the UK [3]–[5]. Most crops are treated with one or more insecticide, leading to a potential conflict between the need to manage insect pests and the risks of harming pollinator populations.

One group of insecticides in particular, the neonicotinoids, have been suspected of contributing to declines in bees [6]–[9]. These chemicals are among the most widely used pesticides globally, and are routinely used as seed dressings for crops such as oilseed rape and sunflower. Imidacloprid alone is registered for use on over 140 crops in over 120 countries [10]. In the UK, use of neonicotinoids has increased year on year since 1994, with ∼80,000 kg applied to >1.2 million hectares of crops in 2010 [11].

Neonicotinoids bind to the postsynaptic nicotinic acetylcholine receptor (nAChRs) in insects, causing over stimulation of the central nervous system leading to paralysis and death [12].They are systemic, rendering the growing plant toxic to insect herbivores and thus reducing or removing the need to apply aerial sprays of insecticides. However, the compounds occur at low levels (0.7–51 µg L^−1^) in both the nectar and pollen of the crop when it flowers, so they are likely to be consumed by pollinators. A recent meta-analysis based on 13 studies of the impacts of imidacloprid on honeybees found that field-realistic doses under laboratory and semi-field conditions had no lethal effects but reduced colony performance by 6 to 20% [13]. It is now becoming clear that subtle sublethal effects of pesticides as described by Desneux et al. [6] can have profound implications at the colony level. For example Henry et al. [9] showed that honeybees, after being fed with sublethal doses of thiomethoxam, had a lower chance of finding their home colony than control bees. Whitehorn et al. [8] simulated exposure of bumblebee colonies to a crop of flowering oilseed rape treated with imidacloprid and describe reduced nest growth and an 85% drop in queen production compared to control colonies. Gill et al. [14] found that bumblebees exposed to imidacloprid exhibited a reduced foraging efficiency under field conditions, particularly when collecting pollen, while Schneider et al. [15] describe reduced foraging activity following exposure of honeybee foragers to low levels of either imidacloprid or clothianidin. These studies suggest that neonicotinoids may indeed be having significant impacts on bees, although how this translates into population-level effects is not clear [16]. However, studies to date have largely used experimentally dosed bees where the bees were unable to avoid feeding on the insecticide. If pollinators are attracted to or repelled by treated crops (compared to controls), then their level of exposure to neonicotinoids could be higher or lower than expected. Previous studies suggest that honeybees avoid imidacloprid in nectar, but the doses used were higher than naturally encountered in nectar of treated crops [17]. Here we describe a simple experiment to assess whether the neonicotinoid insecticide imidacloprid increases or decreases attraction of flying insects to coloured pan traps.

## Materials and Methods

A total of 50 bright yellow rectangular pan traps (17×11×4 cm) were randomly distributed as 25 pairs in a ∼4 ha area of unmanaged neutral grassland at the University of Stirling, Scotland (56° 08′ 39″ N, 3° 54′ 45″ W). The area has not been managed other than by occasional mowing for in excess of 10 years, and contains a diversity of flowers including, for example, *Cirsium arvense, Taraxacum officinale, Potentilla reptans, Lathyrus pratensis and Hypochaeris radicata,* Pan traps are a standard technique for sampling flower-visiting insects, and yellow is generally the most effective colour to attract large numbers [18]. However, pan traps attract also catch many other flying insects, and they do not contain sugar or floral scents found in flowers, so caution is needed in interpreting patterns of capture as reflecting insect behaviour on real flowers. Trap pairs were situated 1 m apart, and were placed on the ground. All traps were filled with ∼250 ml of water plus two drops of detergent (which breaks the surface tension and so improves the catch). In addition, a low concentration of imidacloprid (analytical grade, Sigma-Aldrich, USA) was added to one of each pair at random. Traps were emptied and refilled every 48 h, since imidacloprid can be rapidly degraded by sunlight. All arthropods were preserved in 60% ethanol until they could be identified to Order and counted.

Three consecutive trials were performed, using different concentrations of imidacloprid. Each trial lasted 14 days, so that 7 repeat samples were collected from each trap. These were pooled into a single sample for analysis.

Trial 1: Control versus 1 µg l^−1^ imidacloprid, 19 June–2 July 2012.

Trial 2: Control versus 0.1 µg l^−1^ imidacloprid, 5–18 July 2012.

Trial 3: Control versus 0.01 µg l^−1^ imidacloprid, 20 July–2 August 2012.

For analysis, data were pooled from all seven two-day periods within each of the three consecutive trials to give a single value for the number of arthropods per trap per 14 days. Data were analysed separately for each trial and for Diptera, Coleoptera and Araneae, using Generalized Linear Models in IBM SPSS Version 19 with Poisson errors (nine GLMs in total). Numbers of other invertebrates were too low for meaningful analysis. Treatment (imidacloprid versus control) was the only predictor (explanatory factor) included in each model.

## Results

**Figure 1:**
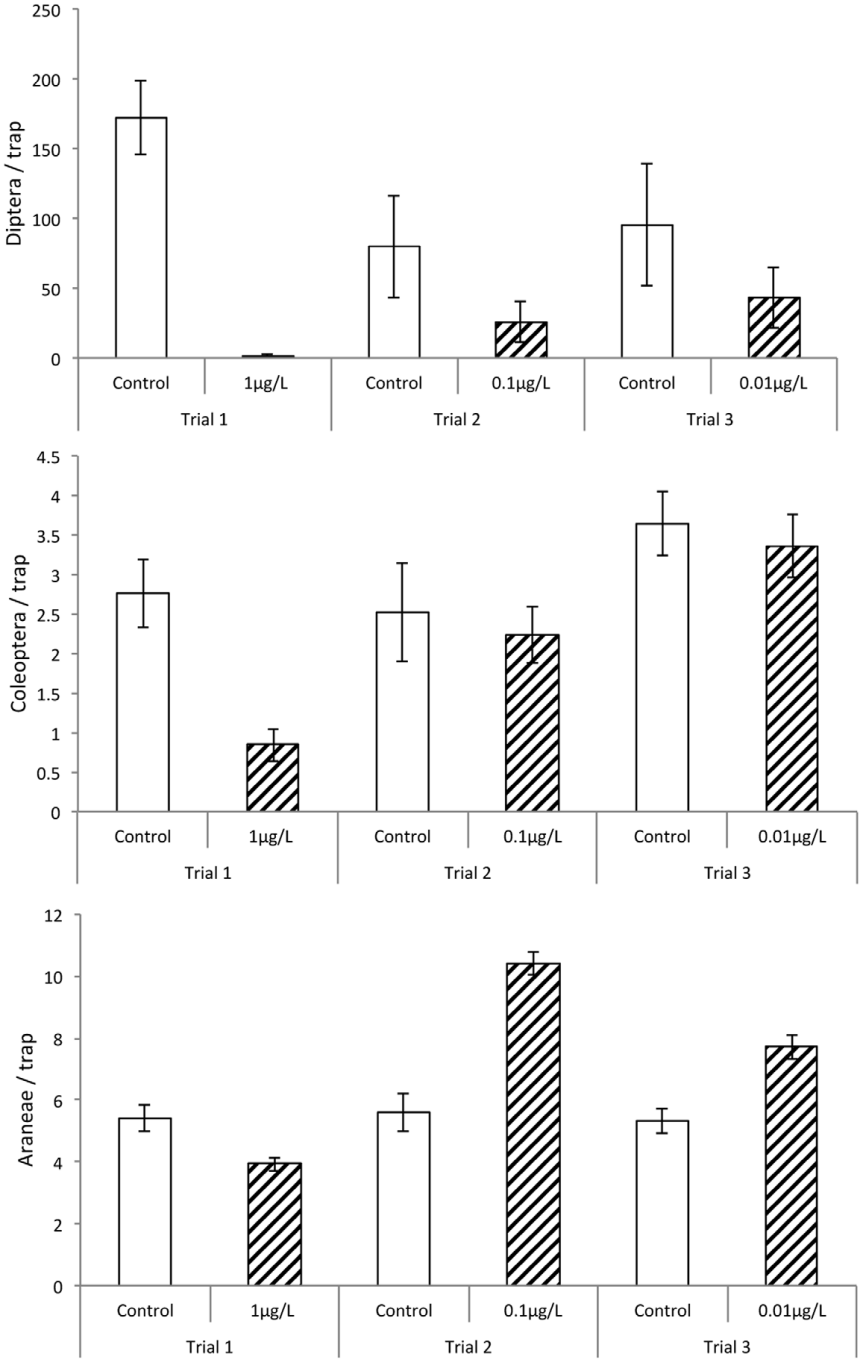
Mean numbers of arthropods (± SE) caught per trap per 14 day sampling period (n = 25). A) Diptera; B) Coleoptera; C) Araneae.

The bulk of the 11,967 arthropods caught in the pan traps belonged to the Diptera (87.1%), including Syrphidae such as *Eristalis tenax* and *Episyrphis balteatus*, and also Chironomidae, Tephritidae, Tachinidae and Calliphoridae. The other arthropods consisted primarily of Araneae (8.0%, mainly Lycosidae, Araneidae and Linyphiidae) and Coleoptera (3.2%, mainly Nitidulidae [pollen beetles], Cantharidae and Scarabaeidae), with the remainder (Lepidoptera, Odonata, Hymenoptera, Hemiptera, Thysanoptera, Orthoptera) comprising just ∼1.7% of the total catch (Figure 1).

The number of Diptera caught was strongly reduced when imidacloprid was present, at all three concentrations, although the repellent effect became markedly weaker at lower concentrations (Figure 1a, Table 1). Coleoptera exhibited a similar response at the highest concentration used (1 µg L^−1^), and there was no discernible effect at lower concentrations (Figure 1b, Table 1). Spiders also exhibited a repellent response at 1 µg L^−1^, but exhibited significant attraction to imidacloprid at lower concentrations (Figure 1c, Table 1).

**Table 1:** Wald χ^2^ values and significance for the effects of imidacloprid on catch of arthropod taxa (D.F = 1 in all cases).

Trial	Control versus 1 µg l^−1^	Control versus 0.1 µg l^−1^	Control versus 0.01 µg l^−1^
Diptera	675P<0.001	613P<0.001	474P<0.001
Coleoptera	22.8P<0.001	0.411n.s.	0.280n.s.
Araneae	5.83P = 0.016	35.4P<0.001	10.9P = 0.001

## Discussion

Overall, Diptera, Coleoptera and Araneae exhibited avoidance of pan traps containing 1 µg L^−1^ imidacloprid compared to controls, with Diptera in particular exhibiting a very strong response. This avoidance response remained detectable in dipterans when using concentrations of imidacloprid as low as 0.01 µg L^−1^, below the lower limit of detection using most commonly available analytical methods [e.g. 19]. Our data demonstrate that arthropod sensory systems are highly sensitive to this compound. Strong repellent effects of low concentrations of a pesticide across a broad range of insects are unexpected, but perhaps may be the result of the widespread use of neonicotinoids in farmland and gardens over the previous twenty years leading to powerful selection for their avoidance.

Concentrations of imidacloprid similar to and sometimes considerably exceeding the highest level used here have been found in nectar and pollen of treated crops [20]. Of course caution is needed in interpreting the implications of our results for real flowers. Our catch contained a broad range of (predominantly flying) insects including some well-known pollinators such as Syrphidae, but also many other species whose status as pollinators is not known *A* key question is whether insects are repelled in a similar way by neonicotinoids when they are present in nectar and pollen, or indeed when incorporated into the vegetative tissue of plants. Nectar contains scents which may mask the presence of neonicotinoids, and it provides a sugar reward which may motivate insects to visit flowers despite the presence of neonicotinoids. To our knowledge, marked avoidance of neonicotinoid-treated flowering crops by insect pollinators has not been reported, but equally we can find no evidence that some avoidance does not occur. In lab studies, neonicotinoids have been found to reduce foraging rates of bees in situations where the bees had no alternative choice of food [e.g. 21, 22 and studies cited in 13], but how bees behave when given a choice is not clear, and no previous studies have been carried out on either Diptera or Coleoptera. Indeed, almost nothing is known of the impacts of neonicotinoid pesticides on the behaviour of non-target insects other than bees.

If insects do avoid visiting the flowers of treated crops, this could impact adversely on yield, depending on the strength of the behavioural response and the abundance of pollinators. In intensive systems where large areas of monocultures are grown, there is already some evidence for pollinator limitation of yields [23], and this could be exacerbated if pesticides repel pollinators such as syrphids. Conversely, avoidance of treated crops would reduce exposure of pollinators to pesticides and so could benefit pollinator populations, provided of course that there are alternative forage sources available to them in the landscape.

There has been some debate as to whether the brief glut of food provided by flowering crops such as oilseed rape is beneficial or harmful to populations of pollinators such as bumblebees. The evidence so far is mixed [e.g. 24–27], but these studies have not examined or considered the role pesticides might play in mediating the benefits of mass-flowering crops. Simple experiments offering a choice of treated and untreated crops to a range of pollinators could readily establish whether our results translate into avoidance of real flowers, and whether this is confined to Diptera and Coleoptera or whether it also occurs in better-studied groups such as bees. Additional studies are also needed to establish whether wild pollinators other than bees suffer adverse effects from exposure to neonicotinoids in farmland. In general, it is remarkable how little we understand about the environmental toxicology of this widely used class of insecticides.

Our pan traps were intended to catch pollinators, but being placed on the ground they also caught spiders, mainly Lycosidae. Surprisingly, spiders appeared to be attracted to the intermediate and low concentrations of imidacloprid (0.1 and 0.01 µg L^−1^). Since spiders do not feed on plant material, attraction to crops treated with neonicotinoids (if this occurs) might result in spiders finding themselves in places with few insectivorous prey. There appear to be no field studies of the effects on predatory arthropods of consumption of prey containing low concentrations of neonicotinoids, and this may be an area deserving investigation.
